# Integrating biogeography and behavioral ecology to rapidly address biodiversity loss

**DOI:** 10.1073/pnas.2110866120

**Published:** 2023-04-05

**Authors:** Katharine A. Marske, Hayley C. Lanier, Cameron D. Siler, Ashlee H. Rowe, Laura R. Stein

**Affiliations:** ^a^Department of Biology, University of Oklahoma, Norman, OK 73019; ^b^Sam Noble Oklahoma Museum of Natural History, University of Oklahoma, Norman, OK 73072

**Keywords:** climate change, extinction, biogeography, behavioral ecology, convergent science

## Abstract

Addressing climate change and biodiversity loss will be the defining ecological, political, and humanitarian challenge of our time. Alarmingly, policymakers face a narrowing window of opportunity to prevent the worst impacts, necessitating complex decisions about which land to set aside for biodiversity preservation. Yet, our ability to make these decisions is hindered by our limited capacity to predict how species will respond to synergistic drivers of extinction risk. We argue that a rapid integration of biogeography and behavioral ecology can meet these challenges because of the distinct, yet complementary levels of biological organization they address, scaling from individuals to populations, and from species and communities to continental biotas. This union of disciplines will advance efforts to predict biodiversity’s responses to climate change and habitat loss through a deeper understanding of how biotic interactions and other behaviors modulate extinction risk, and how responses of individuals and populations impact the communities in which they are embedded. Fostering a rapid mobilization of expertise across behavioral ecology and biogeography is a critical step toward slowing biodiversity loss.

The dual crises of anthropogenic climate change and biodiversity loss are quickly eroding the biosphere’s ability to maintain conditions necessary for human flourishing and societal stability (1–4). While the looming threat of climate change has served as a backdrop to the scientific careers of a generation, the dire impacts of biodiversity loss on humanity are only recently coming into focus (1, 5–7). Both have recently been elevated to national and international priorities due to climate change’s increasingly visible consequences and growing awareness of the magnitude of biodiversity loss, but the window for taking action to prevent the worst impacts is quickly closing (3–5, 8). Rapid transdisciplinary action across all areas of expertise (e.g., scientific, social, economic) is needed to stem this crisis; balancing the provisioning of resources to human populations (infrastructure, food, and water) with climate change mitigation and biodiversity conservation will require support from diverse scientific perspectives.

Decisions regarding land use are emerging as a key focus for trade-offs between provisioning human populations, climate change mitigation, and biodiversity conservation, and thus represent an important focus for transdisciplinary collaboration (9–11). Globally, habitat loss is one of the main drivers of extinction risk (5, 12), with land-use transformation for agriculture, particularly in the tropics, as a leading driver of both habitat loss (13) and diminished suitability of adjacent intact environments (14). The direct human pressure on species and their habitats is exacerbated by climate change, which impacts extinction risk directly by pushing species closer to their physiological limits (15, 16), and indirectly by altering food resources (17, 18). Habitat quantity and quality are also affected by our responses to the growing climate threat, such as wind, solar and wave farms, resource extraction for battery creation, carbon sequestration in biomass, or ecosystem-based strategies for climate adaptation (9). Finally, areas currently set aside for biodiversity conservation or provisioning of ecosystem services are typically based on current distributions of focal habitats, species, or species interactions but may not afford long-term protection or provisioning under changing climates as species’ ranges shift (19, 20), with potentially complex economic and political consequences (e.g., ref. 21). Thus, approaches that stabilize the global climate are inextricably intertwined in our efforts to stem the tide of biodiversity loss and must be considered jointly as we work to fulfill the needs of human societies (9).

Given the sweeping impact of these crises, the complexity of interventions, and the narrowing window for the most effective response, developing habitat-based solutions for biodiversity conservation in a rapidly changing biosphere is essential. However, identifying solutions that prevent large-scale extinction requires addressing critical questions about biodiversity dynamics that—despite widespread interest—have proven challenging to answer thus far:

1) What factors determine whether species will be able to move geographically, adapt in situ, or face increased extinction risk in response to changing environmental conditions? Across species, what factors modulate different outcomes?
2) What are the impacts of individual species (i.e., due to their functional role, local abundance, unique genetic variants, or local adaptations) on ecosystem function? Which species, if lost, would trigger a collapse of critical ecosystem services?
3) How do we rapidly scale up from traditional experimental approaches and existing data to address these questions, so that results may be translated broadly across taxa and geographic regions?

Thus, a core scientific challenge is navigating differences in geographical scales and levels of biological organization—from understanding the role of variation in responses to change within and among populations, to the implication of these responses at the species and community level—to predict the chances of persistence of all levels of biodiversity (genetic, species, phylogenetic, functional) in the available habitats of the future. Different aspects of this challenge tend to be the foci of separate biological disciplines, each with the capacity to contribute unique insights into the questions above.

We argue that an urgent integration of biogeography [the study of how and why biological diversity varies across the Earth, (22)] with behavioral ecology [the study of the evolution of behavior in relation to ecological pressures, (23)] is critical to answering these questions. Together, they unite the large-scale perspective of how environmental gradients, evolutionary history, and extinction have shaped species’ distributions (22) with the population- and individual-level perspectives of how species respond to changes in their immediate environment (24) (Fig. 1). Both disciplines provide independent, complementary insights into how species respond to change by explicitly considering the evolutionary and ecological context in which species occur (22), and how environmental variation can shape species’ responses to different stimuli (25)—perspectives which are critical to leveraging existing biodiversity knowledge into predictive frameworks for how biodiversity will respond to environmental change, and where habitat conservation can be most effective.

**Fig. 1.**
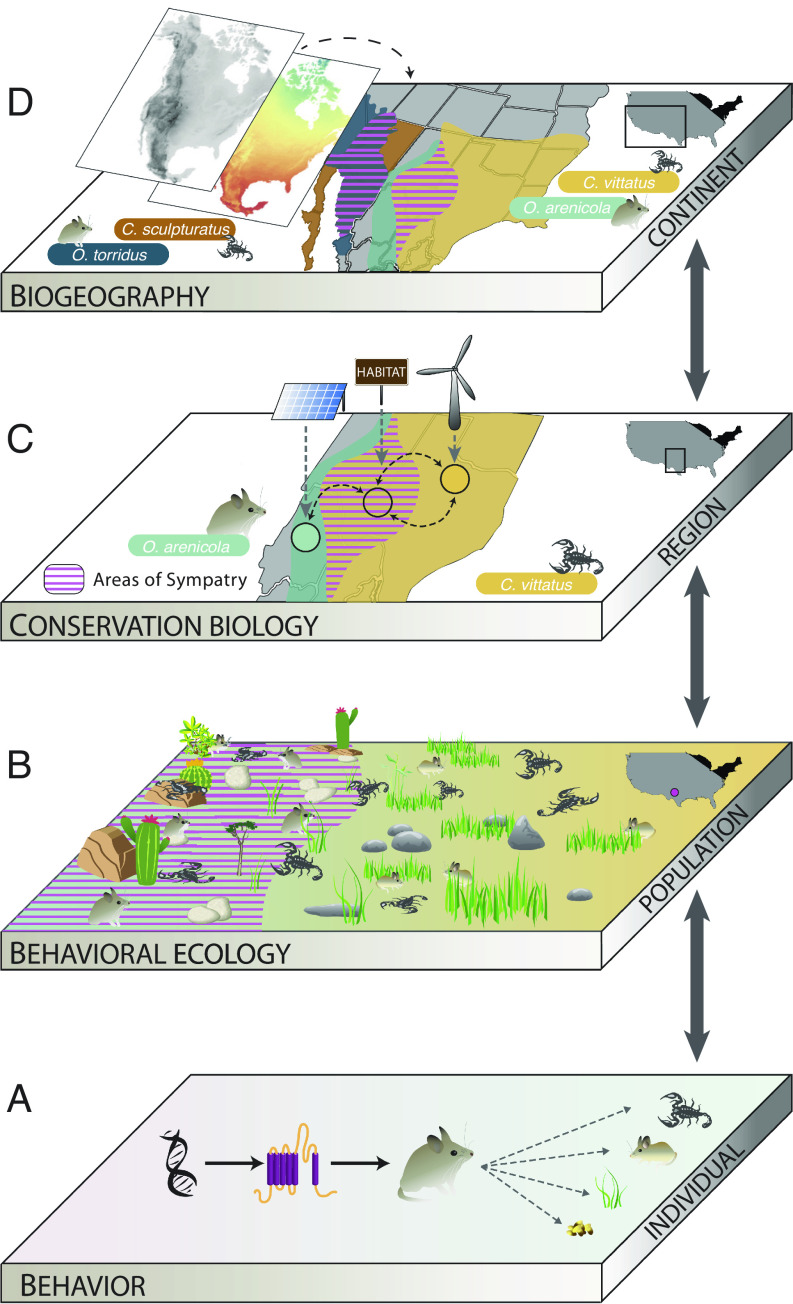
The coevolutionary grasshopper mouse (*Onychomys* spp.) and bark scorpion (*Centruroides* spp.) system in the Sonoran and Chihuahuan deserts of North America (26, 27) illustrates how integration of biogeography and behavioral ecology can shed critical insights into species’ persistence under climate change and habitat loss. (*A*) Variation at the molecular level, such as changes in voltage-gated sodium channels in grasshopper mice that impart toxin tolerance to coevolved scorpions, contributes to predatory behaviors such as prey selection and risk assessment, which shapes (*B*) species abundance within populations and communities, influencing (*C*) persistence within and dispersal and gene flow among regional populations, the level at which most habitat designations and land use decisions are made. (*D*) These dynamics occur across a gradient of abiotic conditions (e.g., elevation and mean annual temperature, as shown in the *Inset* maps, *Upper Left*, based on data from adaptwest.databasin.org), which interact with the biotic environment to delimit species’ geographic ranges. Models of species’ persistence that integrate dynamics across these scales will improve predictions of the effectiveness of habitat-based biodiversity conservation.

Biogeography and behavioral ecology are both highly integrative, allowing the translation of the findings of other research disciplines into an explicitly ecological and evolutionary context. This translation enables the prediction of potential consequences of environmental change. Numerous disciplines (e.g., evolutionary biology, physiology, genetics, and neuroscience) generate data that are relevant to such predictions (e.g., how the molecular and neurological bases for important behaviors contribute to how species interact with their a/biotic environment), but a behavioral ecology perspective translates those findings into a real-world context (Fig. 1). Similarly, dynamics studied by behavioral ecologists, such as the circumstances that make behaviors adaptive or that underpin behavioral plasticity, are linked to the geographic context in which species and communities occur, including variation in climate or topography, or coevolution with species in the same region. Biogeographers, who include macro- and paleoecologists, phylogeographers, and systematists, and utilize data products from physical geography, oceanography, and climate science (among others), are well positioned to provide the geographic contextualization that is essential to translating individual- and population-level responses into regional to global predictions of species’ persistence and global extinction risk.

All of this points to a critical feature of transdisciplinary biogeography of behavior research that is absent from the other sets of collaborations outlined above: capacity for translation of biological patterns and eco-evolutionary processes across taxonomic and geographic scales (Fig. 1). In both disciplines, observations are often of individuals (e.g., behaviors, particular traits or genotypes, or species’ presence/absence from a locality), but the consequences of these findings are scaled to describe impacts within and between populations (behavioral ecology), to the level of species’ geographic ranges (biogeography) and across the communities of codistributed organisms in which focal taxa are embedded (both disciplines). Thus, both disciplines already consider the implications of their results across geographic scales, which is critical for predicting whether species can persist within their current habitats, move to follow optimal conditions, or risk extinction. Our goal with this perspective is to encourage innovative, transdisciplinary research that builds on the strengths of these perspectives, focused on the urgent challenge of preventing biodiversity loss in the face of rapid climate change.

## Both Disciplines Contribute Perspectives Critical to Biodiversity Conservation, but Could Achieve More in Synergy

As environmental conditions change, the extent to which species will be able to track favorable conditions or adapt to disturbances within their current range is critical to their persistence and the ecological services they provide. Ranges are the geographic realization of each species’ ecological niche, restricted to regions the species can access via dispersal (28, 29), and contain the habitats in which species carry out their ecological functions (30). They are the fundamental unit of biogeography (31), which investigates the processes underlying the geographical distribution of species richness (32) and genetic variation (33, 34), the evolutionary distinctiveness of regional biotas (35), and differences in local species composition among communities (36), as well as how these patterns have been shaped by past climatic change (37, 38) and human activity (39, 40). With its emphasis on the factors governing species’ ranges, biogeography provides the context for why species exist where they do, and the methods to estimate where they are likely to exist in the future.

Biogeographers have long sought to predict the impacts of climate change on biodiversity, including using forecast methods to predict the locations of species’ ranges under future climate conditions (41, 42) and querying historical distributions for insights on range dynamics during past climate change (43, 44). Biogeographic approaches have also yielded critical insights into potential interactions among threats by mapping the extent to which land use and other factors (e.g., diseases or invasive species) overlap geographically with climate change risk (13, 45, 46), and contributed to frameworks for designing protected areas given different strategic aims (47). However, one persistent challenge within the subdiscipline of conservation biogeography (48, 49), and biogeography in general, is the limited information on species’ distributions, evolutionary histories, and traits that govern how species interact with their environment (50), and how these factors underpin species’ geographic ranges, even in the absence of rapid climate change.

How species behaviorally interact with their biotic and abiotic environment is a critical component of whether habitats designated for biodiversity conservation will succeed in allowing either persistence or successful movement to new habitats (51–53). These questions are the purview of behavioral ecology, which focuses on the evolution and function of behaviors and how they are transmitted across generations and among populations (23). Thus, behavioral ecology captures key predictors of species’ resilience (51), such as the extent to which behavioral traits can result in novel innovations transmitted across populations (54) or a poor fit between a species’ ecological niche and local conditions (55). The emerging field of conservation behavior (56) has already led to successful interventions for some of the most endangered species on the planet, including predator training for black-footed ferrets (57) and use of sex allocation theory to optimize conservation strategy for New Zealand kākāpō (58). As the climate and biodiversity crises imperil more species, conservation interventions built in synergy with behavioral data will be critical to minimizing species-specific risks to changing environments, particularly in the face of habitat modification and other extinction drivers (51–53).

Behavior shapes species’ persistence and resilience because it is often an individual's first response to environmental change (24)—the *how* and *why* organisms do what they do to survive and reproduce (23). It is thus critical to identify the behaviors that have a strong influence on population and range dynamics to effectively predict how species will respond to climate change. Apart from behaviors that are directly linked to movement and range expansion (e.g., dispersal, migration, habitat preference, personality) (59, 60), several associated with fitness (reproduction: courtship, mating systems, mate choice; survival: foraging, antipredator) or that mediate interactions among community members also shape population-level persistence (61). The degree of variation among individuals across these behavioral traits (59, 60, 62), and the extent to which plasticity modulates individual and community responses to change (63–65), will also impact whether habitats remain suitable. However, the extent to which behaviors are contemporarily adaptive is contingent upon the current environments in which individuals and populations occur (25, 66, 67). Thus, without including biogeographical perspectives in the study of behavior, we may miss patterns of behavioral diversity that provide clues to the mechanisms allowing species to adapt to change (25, 67), behaviors that are critical for maintaining interaction networks under environmental stress (67), or conditions under which organisms might succumb to evolutionary traps (68).

Together, biogeography and behavioral ecology unite a suite of diverse methods and large networks of additional collaborations to yield critical insights about land use for biodiversity conservation, including the challenge of translating biodiversity impacts across geographic and taxonomic scales that are typically addressed by separate disciplines (Fig. 1). Below, we highlight a few areas where this transdisciplinary research agenda can push beyond the traditional limits of each to improve our predictions of biodiversity responses to environmental change, make inroads into addressing the questions above, and advance our ability to inform practical decisions about where biodiversity conservation may be most effective.

## The Behaviors of Individuals Define Each Species’ Ecological Niche and Geographic Range

Biogeographic studies typically consider two main factors which limit species’ geographic ranges: abiotic factors, such as climatic or dispersal barriers (28), and interactions between species (67). Both factors have explicit behavioral components, ranging from how species use habitats (69) and disperse across the landscape (61, 62), to the behavioral traits which mediate species interactions, the outcomes of which impact range utilization and the locations of range margins (70–72). These behaviors may directly impact species’ ability to respond to climate change: For example, bird communities in the Mojave Desert ecosystem have collapsed over the last century (73), while small mammal communities have remained largely stable, likely due to their use of burrows and other microhabitat features to escape the heat (15). For these and other species whose ranges exist at the edges of their physiological tolerance, key behaviors may determine whether species are able to persist under climate change (69). Behavioral responses to the impacts of climate change may also affect species through their participation in biotic interactions: For example, observations of aggressive encounters among Indo-Pacific reef fishes before and after the 2016 global coral bleaching event revealed a breakdown in competitive interactions among butterflyfish (*Chaetodon* spp.), which typically defend habitat patches within reefs from both con- and hetero-specifics (17). Where such behaviors mediate species coexistence, short-term response to depleted food resources may signal the beginning of long-term changes in local abundance, dispersal among reefs and composition of reef fish communities (17). These examples show how insights into the behaviors that facilitate species’ local persistence and mediate species interactions are key to predicting range dynamics across changing habitats (74, 75) and the suitability of particular habitats for long-term biodiversity preservation.

The importance of abiotic conditions versus biotic interactions in limiting species distributions varies at different range margins and across the range (74–77), influencing the extent to which species will be able to shift their ranges in response to climate change or adapt to novel conditions in situ (78, 79). Numerous tools facilitate the incorporation of abiotic drivers of species distributions—largely climate—into biogeographic models of species’ responses to change. In particular, species distribution models (SDMs; also called ecological niche models) link georeferenced observations of occurrence with climatic data to estimate species’ potential ranges under past, present, and future climate scenarios (43, 80). These models, which can be quickly executed for thousands of species, have provided clear warnings on the potential impacts of climate change on the distribution of biodiversity (41, 44), the political complexity of conservation actions needed to save it (81), and the potential consequences of a climate-driven redistribution of biodiversity on our own well-being. For example, a recent study used SDMs for >3,800 mammal species to predict where climate-driven range shifts and land use changes are likely to increase opportunities for disease spillover by bringing formerly isolated viral hosts into geographic overlap (82). The power of these methods comes from their simplicity, in that they can generate predictions even for species for which little is known beyond their distribution; however, this also limits the ability of SDMs to generate precise, scalable predictions of species’ ranges in the absence of additional data [e.g., on population dynamics (83), behaviors (69), or biotic interactions (72)].

For species with existing behavioral data, integration of these behaviors into geographical models can provide immediate, realistic estimates of risk. Pikas represent a prime example: Alpine adapted and sensitive to high temperatures, pikas have long been considered the “canaries in the coal mine” of climate change (84). American pikas (*Ochotona princeps*) show evidence of significant range contraction based upon both historical resurvey data (85) and SDMs, which predict drastic declines in suitable habitat under warmer climates (86). Yet, studies of behavioral plasticity have revealed mechanisms for tolerating higher temperatures, including modulation of foraging time (87), and there is skepticism regarding the magnitude of risk pikas face from climate change (88). Applying mechanistic models that included behavioral buffering (e.g., changes in surface activity time), in addition to abiotic predictors, has bridged this disconnect, resulting in an improved outlook with up to 18% less habitat loss (89). Here, the integration across behavioral and biogeographic approaches improved risk projections, producing better-informed population projections and management needs for this iconic species and alpine habitats.

Predicting the extent to which biotic interactions may hinder or help species responses to environmental change is trickier than predicting the distribution of species’ realized climatic niche because it requires understanding which interactions have already been important in shaping the ranges of species and the extent to which they are likely to continue (67). As these data are not known for many species, biogeographic approaches often leverage cooccurrence to make inferences about biotic interactions (76). Two recent avian examples used the orientation of ranges (71) or individual occurrences (90) to demonstrate that species’ distributions are limited by interspecific competition, which will likely impact species’ abilities to disperse in response to environmental change, in ways that cannot be directly foreseen by climate-based models, although the net result may be similar [e.g., upslope shifts in tropical montane bird communities (90)]. These findings—and their implications—support previous SDM-based studies which also demonstrate the impacts of biotic interactions on species’ distributions (91). However, geographically distributed data on the most relevant biotic interactions remain difficult to obtain for most single-species models (72, 76).

One promising alternative to using cooccurrence to infer the impacts of biotic interactions might be to focus on well-characterized systems with respect to biotic interactions, even if they are not yet widely geographically sampled, and build outward. One type of system that might yield powerful results when leveraged in a climate change context is geographic mosaics of coevolutionary relationships (67), which are likely to constrain multiple species’ responses to environmental change. For example, predatory grasshopper mice (*Onychomys* spp.) and their bark scorpion prey (*Centruroides* spp.), iconic denizens of the North American deserts, exhibit a geographic mosaic of sympatric and allopatric populations across arid landscapes (92) (Fig. 1). The variation in the biogeographic histories of scorpions and mice has shaped the strength and duration of coevolutionary pressure across the ranges of both taxa, resulting in genomic and behavioral differences among populations related to both the toxins present in scorpion venoms and toxin resistance in grasshopper mice (26, 27). Scorpions are a key prey item during seasons when other arthropods are scarce, and so toxin resistance matching may be important in shaping persistence of grasshopper mice, with populations facing decreased probability of survival with the loss of key members of their interaction network (67). With climate change reducing variation in the seasonal monsoons that separate deserts—each with its own coevolved scorpion-mouse community—the potential for novel interactions between bark scorpion species and naïve grasshopper mouse populations is increasing.

We propose a two-pronged approach to explore the impacts of climate change in this system: First, range maps or species occurrences enable the building of baseline predictions of range dynamics under climate change, which can be validated against the distributions of biotic interactions (including predator–prey and among sister species) and variation in the strength of reciprocal selection (if known) in different environments across the geographic mosaic. An important output of these models would be to estimate the sensitivity of range predictions for individual species on the extent to which biotic interactions are incorporated. Second, these model-based approaches might identify key field locations to monitor for early indications of changes in geographic overlap or interaction strength among species to further refine predictions of range shifts and changes in community composition (93). Both steps of this process, leveraged with novel genomic resources (94), can yield critical insights into how interaction networks are formed, the flexibility of networks under change, and the extent to which that flexibility is impacted by variation in a/biotic environments versus coevolutionary constraints.

## Leveraging Model Systems: Behavioral Plasticity in Key Species May Determine the Ability of Communities to Adapt

While biogeographic methods include approaches to address the lack of range-level behavioral observations for most species, one drawback is that behavioral traits (including biotic interactions) are often treated as relatively set (i.e., all individuals of a species share the same traits, which will not change under future conditions). This may not reflect how variation in behavior, including standing variation or as a result of plasticity, may shape the ability of species to adapt, acclimatize, or relocate in response to change (24, 55). These limits to characterizing species’ behavioral flexibility are likely to lead to over- or underestimation of their abilities to adapt to changing environments, particularly where climate change and habitat disruption or other threats interact (see section on *Habitat Loss*, below). In general, phenotypic plasticity—and heritable variation in plasticity among individuals in different environments (95)—will likely prove critical in determining species’ responses to environmental change. For example, work in *Caenorhabditis elegans* (96) and *Acropora cervicornis* (staghorn coral) (97) have identified heritable correlations of genotype-by-environment interactions with temperature, suggesting heritable variation in plastic responses to the environment that may be an important predictor of niche adaptation and, in turn, range expansion or maintenance. Here, we focus on behavioral plasticity as a foundational component linking genes, behavior, and environment to broad-scale range dynamics and extinction risk, due to its consequences for both the focal species and the communities in which they occur.

Behavioral plasticity, or the ability of organisms to flexibly adjust their behavior in response to the environment, has been proposed as a major mechanism for predicting species survival in the face of climate change (55, 98) and is governed by gene-by-environment interactions. Genetic variation in habitat preference (99), group size preference (100), and behaviors such as aggression (101) has been shown to differ across populations, and these are important mediators of dispersal, habitat selection, and social niches. While such behavioral flexibility can help species cope with environmental change, including temperature shifts [e.g. refs. 63, 102, and 103], it may not be a consistently strong predictor of extinction risk (98), and in some cases may contribute to the heightened risk of the focal species (104) or those with which it interacts. Thus, simply quantifying the degree of behavioral plasticity within a species is insufficient to understanding its impact on climate change response. To date, the vast majority of theoretical and empirical work on plastic responses to climate change have focused on within-generational plasticity (24). However, transgenerational plasticity (parental ability to convey information across generations) may also play a key role in adaptation to rapidly changing environments (65). Transgenerational plasticity might be especially relevant in understanding the potential for species to persist in situ during climate change because it can buffer populations against the immediate effects and provide time for genetic adaptation to catch up (64, 65). Alternatively, if environments are changing too rapidly or less predictably, it may be maladaptive by preparing offspring for an environment they will never encounter (65, 105).

Empirical work has implicated transgenerational plasticity in response to rapidly changing environments, and such plasticity may be responsible for potential “invisible barriers” to range expansion (106). Transgenerational plasticity may be particularly important for sessile broadcast spawners, where parents have little opportunity to provide information to offspring about the environment, and offspring behavior is relatively limited to habitat selection. Paternal density of the ascidian *Stylea plicada* improved offspring survival under conditions that matched those of their fathers, implicating sperm-mediated effects in offspring habitat selection and survival (107). Across three species of corals, parental depth influenced offspring success such that offspring were less successful when mismatched with their parental environment (106). With depth closely tied to temperature (106), offspring preference and success for parental environments have the potential to create “evolutionary traps” by placing corals in habitats outside their thermal tolerance. Altogether, recent work in broadcast spawners that do not show obvious “intentional” behaviors suggest complex interactions between parental experience and species that are important to consider when identifying areas for habitat protection or transplantation.

Behavioral plasticity of focal species can also impact community-level population dynamics, as is already becoming apparent in studies of threespine stickleback fish (*Gasterosteus aculeatus*), which are widely distributed in the northern hemisphere and are rarely targeted by conservation strategies due to their historical abundance, despite their ecological importance in food webs (108). While some populations thrive under eutrophication and warming temperatures (109), others are rapidly declining or becoming extinct, particularly at lower latitudes (110). In northern California, continuing drought has reduced flowing streams to still-water environments, increasing temperature and eutrophication, but also increasing interactions between species as available habitat is reduced. Stickleback fathers who encounter predators adjust their parenting behavior, and their offspring show greater antipredator phenotypes (111). Transgenerational plasticity may accelerate the prevalence of antipredator phenotypes in sticklebacks (112), which in turn can influence population dynamics of their sculpin and salmonid predators as these predators adapt to a greater frequency of anti-predator phenotypes. Such indirect impacts of climate change are already being observed in the Baltic Sea, where increases in stickleback populations due to eutrophication and warming have led to decreasing populations of pike and perch that are otherwise resilient to these abiotic changes (109, 113).

We propose that many characteristics that make sticklebacks a model system for evolutionary adaptation (114) (broad geographic distribution, occurrence in diverse communities, rapid colonization, and subsequent local adaptation into freshwater bodies), also make them a candidate model for investigating indirect impacts of climate change on populations and communities that may not yet be considered at risk, across an environmental gradient that is becoming increasingly harsh. Replicated experiments and surveys along this gradient would provide critical insights into how joint changes to the a/biotic environment shape patterns of phenotypic variation, and how these changes impact fish community structure, species richness, and regional metapopulation dynamics (e.g., as drought conditions impact opportunities for dispersal). Here, previous studies of plasticity at different points along the gradient form a critical baseline against which changes in behavioral and community dynamics can be compared.

## Habitat Loss: Testing the Limits of Each Discipline for Predicting Species’ Responses to Change

Climatic disruption of species’ ranges has already begun, with numerous plant and animal communities already experiencing range shift (115, 116). However, the capacity for species to respond to climate change is also impacted by human-mediated habitat loss, fragmentation, and degradation within their ranges or adjacent regions that once offered dispersal corridors. While there are notable examples of species that have successfully adapted to human-dominated spaces (117, 118), far more are currently at risk of extinction due to habitat modification or loss (5, 119); for example, 70% of remaining forest globally is subject to edge effects as a consequence of habitat fragmentation (14). In some cases, a lack of behavioral plasticity impacts species’ persistence even where habitat patches remain: Global mammal tracking data show significant reduction in individual movement in areas with high human activity (120), while Amazonian forest birds, particularly those with complex behavioral associations like army-ant followers, have experienced restricted movement in response to road-building through otherwise intact habitat (121). These negative responses to habitat disturbance suggest that many species will not be able to shift their ranges to follow climate change across increasingly modified landscapes. The largest and longest-running study of habitat fragmentation, in central Amazonia, has revealed a variety of species responses to habitat fragmentation, from loss of species richness among insectivorous birds, bats, primates, and other large mammals in forest fragments, to declines in abundance in birds, bats, and insects as diverse as beetles, flies, butterflies, bees, wasps and ants in forest-edge habitats (122 and sources therein).

Habitat loss adds to the challenge of climate change by reducing species’ access to areas within their climatic niche, reducing connectivity among populations. Together, habitat loss and climate change may profoundly diminish gene flow among and standing genetic variation within populations, limiting species’ opportunities for range maintenance or shift (123, 124), changing the landscape of adaptation (125), and potentially impacting the contributions of individuals and populations to ecosystem processes (126, 127). As ranges diminish or habitat fragments become increasingly isolated, small populations become more susceptible to inbreeding depression and genetic drift, reducing genetic diversity and adaptive potential, which can contribute to further range reduction and population decline [i.e., pushing populations into an extinction vortex (128)]. A recent study of >10,000 georeferenced whole genomes for 20 species suggests that such genetic erosion may already be widespread, even in species not currently threatened with extinction (129). Using the species–area relationship, the authors estimated that localized population extinctions resulting from pre-21st Century land use change have already driven a loss of 10% of global genetic diversity (129). Aggregation of data across population genetic studies also shows warning signs: Urban populations of North American mammals have lower effective population sizes and reduced genetic diversity than their counterparts in more natural environments (130), consistent with the disturbance suggested by the reduction in activity patterns noted above. Where this genetic erosion affects the behaviors and other traits that modulate interactions among organisms, the genetic consequences of environmental change have the potential to shape community-level responses.

Protecting critical habitats to prevent biodiversity loss (including defaunation and genetic erosion) in the face of climate change requires modelling how behavioral nuances are likely to impact persistence of populations in spatially complex constellations of remaining habitat (14, 40). Thus, predicting the impacts of habitat loss on species that are already affected by climate change will test the limits of both biogeography and behavioral ecology. The examples of species’ behavioral responses to habitat fragmentation, above, highlight that despite the predictive strength of biogeographic tools, they will likely be insufficient on their own for predicting how biodiversity will fare in landscapes that have been transformed by humanity. However, for most species, geographically representative data on behaviors and interaction networks, as well as accurate range maps and genetic profiles, will not become available in time to drive the decisions that may determine species’ fates, given the current pace of climate change and habitat transformation. Thus, our challenge is to predict the fate of diverse species, communities, and ecosystems, given the data that already exist or that can be rapidly collected within the next few years. Solutions require mobilizing biogeographers and behavioral ecologists to identify the critical dynamics that link biodiversity patterns across scales, and increased collaboration with ecological modelers and other data scientists to develop innovative tools to unite disparate data types (e.g., spatial grids and points, networks, and behavioral observations), encode complex behaviors, and adopt computational methods that leverage existing knowledge in the face of uncertainty.

Geographically realistic mechanistic models and emerging computational tools like machine learning and artificial intelligence have already shown significant promise in predicting how the impacts of climate change, habitat loss and species exploitation are likely to impact communities. For example, spatially explicit, mechanistic simulations of predator–prey dynamics in size-structured marine food webs showed that trophic interactions limited species’ abilities to shift their ranges under climate change (131). Machine learning methods, which can search for patterns across large, complex datasets, have been used to inform zoonotic disease monitoring efforts (132), to assess threat status for >7,000 species identified as Data Deficient by the International Union for the Conservation of Nature (133), and to model the impacts of extinction on mammal food webs (134). To predict the impacts of animal defaunation on global plant dispersal, one study used machine learning to leverage ~18,000 plant–frugivore interactions from 406 networks, together with trait-based models for seed dispersal based on 2,215 different germination experiments, to infer interactions based on species’ traits (135). They showed that loss of seed-dispersing mammals and birds has reduced the potential for plants to shift their ranges in response to climate change by ~60% globally, with regions differentially impacted by the historical loss of large mammals or where dispersal is currently conducted by species at heightened extinction risk (135). Together, these studies demonstrate the promise of advanced computational methods to make the most of existing biogeographic and behavioral data by broadening the pool of species and the nature of biodiversity dynamics for which we can predict the impacts of environmental change.

We envision several opportunities where computational innovations that integrate biogeographic and behavioral data can strengthen our ability to predict future biodiversity responses to global change at geographic scales relevant to conservation. Predictions based on well-studied interactions can be extended to under-sampled geographic regions using machine learning based on phylogenetic relationships, behavioral and functional traits, environmental conditions, and documented patterns of coexistence within specific environments. For well-studied behavioral systems, machine learning might aid in identifying critical individual behaviors or biotic interactions that impact species persistence. Machine learning and mechanistic models, together, can also be used to predict the types of environmental change most likely to impact a local network or community given its constituent species. Similar search methods applied to genomic resources could detect levels of neurogenetic variation and plasticity suggestive of behavioral adaptation, which could feed into the types of experimental approaches described for the grasshopper mouse and stickleback systems. Novel approaches to combine disparate data types will significantly expand our ability to identify species most exposed to extinction risk and forecast the impact of their loss on their communities and ecosystems. However, these opportunities will require deliberate efforts to connect people with different types of expertise, and appropriate recognition for diverse contributions, including data provisioning, model calibration, translation of results into conservation recommendations, and communication to diverse stakeholders.

## Translating Research into Action

Above, we highlighted three areas where convergent “biogeography of behavior” research can lead to significant gains in predicting biodiversity dynamics in a rapidly changing world. However, that research will only take us part of the way toward a solution, and it is critical that researchers also contribute to translating this knowledge into meaningful conservation actions. Fostering action requires making data and findings accessible and interpretable across disciplines and strengthening interactions between conservation practitioners and academics. It may also require consideration of how we prioritize and assign credit for collaboration and encouragement of conservation-oriented outcomes of basic research.

One key step forward will be recognizing where opportunities exist to immediately contribute, particularly for researchers who generate data relevant to the biodiversity dynamics described above, but who have not traditionally seen themselves as global change researchers. For biogeographers, this may mean improved communication around areas of uncertainty in geographic models and their potential impact on different conservation applications (136, 137), particularly when translating model results across geographical scales (138, 139). For behavioral ecologists, this means going beyond acknowledging and documenting the impacts of environmental change on species’ behaviors (52), to identifying their significance as a selection pressure for behaviors of interest and developing predictions for when and how we expect behavior to facilitate or constrain adaptation (140). For both, assessing how our data can address the biodiversity data shortfall (50) is an obvious way to contribute. This may include sharing unpublished information on field research methods, where information on search effort may shed insights on regional biodiversity changes (e.g., species not collected despite sustained survey efforts, or chance behavioral observations such as would be documented in natural history notes). Significant components of recognized knowledge shortfalls likely already exist in the form of underutilized datasets available in the literature or housed in natural history collections, and ramping up support for the digitization of these collections and associated field logs or reports, as well as finding mechanisms to share data for specimens that are not housed in collections (141), may significantly advance our understanding of communities and ecosystems experiencing change. Broader access to data on the genetic and neural basis of behavior could facilitate interpreting this information in an ecological context, such as providing clues to levels of plasticity and genetic variation needed for adaptation.

Facilitating the translation of convergent research into conservation actions that benefit biodiversity and society also requires participation across a broad group of scientists. Fundamentally, investigators need to interact across disciplines to address questions across geographic and taxonomic scales, and with conservation practitioners to identify and fill key knowledge gaps that limit our ability to predict species’ responses to change (e.g., ref. 142). Scientific and conservation societies are well poised to build interactive groups among disciplines by deliberately showcasing disciplinary expertise at joint meetings. In the US, the NSF is already playing a role in promoting convergent research focused on climate change (e.g., Organismal Responses to Climate Change). The rapid expansion of integrative training opportunities within academic research units is also a component, preparing students and postdocs to address convergent research questions and build the necessary connections to conservation organizations to foster the skills needed for translational research. Whether cross-domain training opportunities are established with private or nonprofit organizations, or state or federal agencies, these programs already have a boots-on-the-ground view of conservation and information needs.

However, mobilizing widespread action among scientists also requires rethinking whether the incentive structure for scientific discovery is adequate for driving innovation in preventing extinction, including through funding acquisition and how credit is distributed among peers and academic departments. While the NSF has already started to consider funding with a greater emphasis on solutions (e.g., the Partnership to Advance Conservation Science and Practice; Organismal Responses to Climate Change), expanding the scope of basic research across calls to translate results into tangible, applied conservation outcomes could greatly catalyze meaningful activities. Action could be further incentivized in the USA by considering development of biodiversity conservation plans, actions, or knowledge-sharing as potential Broader Impacts (i.e., desirable societal outcomes stemming from funded research) of research-oriented proposals. This would motivate “closing the loop” to bring conservation-relevant research into conservation planning, without requiring new funding programs. In addition, universities need to find approaches to stimulate convergent research, as well as recognize and reward its translation to applied conservation outcomes as research activity. Providing seed funding for collaboration workshops or the initiation of convergence centers around global challenges like climate change and biodiversity loss is a positive move; however, care should be taken to ensure that such opportunities are not restricted to large, well-established research teams, as this limits the potential for a broader, more impactful outcome. Desired outcomes from these collaborations should include translational applications for biodiversity conservation that can be implemented by state, national, international, or non-governmental conservation organizations—innovations that are in the public interest but are unlikely to contribute significantly to revenue.

Given widespread biodiversity loss, the increasingly realized impacts of climate change, and a limited window for making informed decisions, we need an all-hands-on-deck approach that builds on our combined expertise to determine where and how to preserve the diversity that still exists, given ongoing changes in habitat. By deliberately integrating across biogeography and behavioral ecology to address key information gaps on biodiversity responses to environmental change, we can contribute the scientific guidance needed to inform complex decisions on where setting aside habitats to conserve biodiversity might be most effective. These actions have the added benefit of mitigating the impacts of climate change on human populations, fostering stronger linkages among scientists working on these problems, and leveraging emerging analytical methods that will integrate existing data into new predictive frameworks.

## Data Availability

There are no data underlying this work.
